# Drivers of Wetland Conversion: a Global Meta-Analysis

**DOI:** 10.1371/journal.pone.0081292

**Published:** 2013-11-25

**Authors:** Sanneke van Asselen, Peter H. Verburg, Jan E. Vermaat, Jan H. Janse

**Affiliations:** 1 Institute for Environmental Studies, VU University, Amsterdam, The Netherlands; 2 Earth Sciences and Economics, Faculty of Earth and Life Sciences, VU University, Amsterdam, The Netherlands; 3 PBL Netherlands Environmental Assessment Agency, Bilthoven, The Netherlands; University of California, Berkeley, United States of America

## Abstract

Meta-analysis of case studies has become an important tool for synthesizing case study findings in land change. Meta-analyses of deforestation, urbanization, desertification and change in shifting cultivation systems have been published. This present study adds to this literature, with an analysis of the proximate causes and underlying forces of wetland conversion at a global scale using two complementary approaches of systematic review. Firstly, a meta-analysis of 105 case-study papers describing wetland conversion was performed, showing that different combinations of multiple-factor proximate causes, and underlying forces, drive wetland conversion. Agricultural development has been the main proximate cause of wetland conversion, and economic growth and population density are the most frequently identified underlying forces. Secondly, to add a more quantitative component to the study, a logistic meta-regression analysis was performed to estimate the likelihood of wetland conversion worldwide, using globally-consistent biophysical and socioeconomic location factor maps. Significant factors explaining wetland conversion, in order of importance, are market influence, total wetland area (lower conversion probability), mean annual temperature and cropland or built-up area. The regression analyses results support the outcomes of the meta-analysis of the processes of conversion mentioned in the individual case studies. In other meta-analyses of land change, similar factors (e.g., agricultural development, population growth, market/economic factors) are also identified as important causes of various types of land change (e.g., deforestation, desertification). Meta-analysis helps to identify commonalities across the various local case studies and identify which variables may lead to individual cases to behave differently. The meta-regression provides maps indicating the likelihood of wetland conversion worldwide based on the location factors that have determined historic conversions.

## Introduction

Wetland loss and degradation occur worldwide, sometimes at extremely high rates [1]. In the conterminous US, 53% of wetlands were lost between the 1780s and 1980s, with Ohio and California losing 90% and 91% respectively [2]. Estimated wetland loss in different parts of Canada ranges between 65% and 80% [3]. In parts of Europe, Australia and New Zealand more than 50% of specific types of wetlands were destroyed during the twentieth century [4,5]. In Sumatra and Kalimantan, Indonesia, it is estimated that less than 4% of peatland, comprising undisturbed pristine peat swamp forests, remains, while 37% display varying degrees of degradation [6]. Frequently-cited anthropogenic causes of wetland loss and degradation, include drainage for crop production and plantations, wetland conversion for fish production, conversion for logging, peat extraction, construction of canals, dykes, dams and levees, and commercial, residential and industrial developments, e.g., [1,2,4]. Natural causes include sea-level rise, droughts, storms, and subsidence [1,7-9]. Underlying drivers of wetland loss are socio-economic and political factors, such as population growth and financial policies [1]. Most studies reporting wetland conversion are conducted for single locations. A more comprehensive analysis of the most important drivers of wetland conversion at a global scale is currently lacking. 

Processes of wetland loss and degradation undermine the capacity of wetlands to provide valuable ecosystem services to humanity. These include water supply, flood control, carbon storage, maintenance of biodiversity, retention of sediment and nutrients, and recreation [1,5,10]. Such services have both global significance and local value, and there is broad support for their conservation value, e.g., [5].

In land change studies, a distinction is often made between proximate causes and underlying driving forces, e.g., [11]. Proximate causes are human activities or immediate actions at the local level that originate from intended land use and directly impact land cover. Underlying driving forces are fundamental societal processes, such as human population dynamics or agricultural policies, that drive the proximate causes, either operating at the local level, or indirectly from a higher level. To better understand the proximate causes and underlying driving forces of wetland conversion, and the interlinkages between these processes, we did a systematic, worldwide comparative review of case studies of wetland conversion. Such meta-analyses have already been done for tropical deforestation [11,12], desertification [13], agricultural intensification in the tropics [14], swidden agriculture changes in tropical forest-agriculture frontiers [15], and for urban land conversions [16]. Despite the importance and scale of wetland conversion no meta-analysis is yet available. A more generalized understanding of the proximate causes and underlying driving forces of wetland conversion can be helpful in designing national and global-scale policies and governance options, which will halt further loss of wetlands.

A common problem in the meta-analysis of qualitative and narrative case studies is the comparability of the driving factors mentioned, and the limitations on extrapolating the results due to their qualitative character. We have, therefore, used georeferenced data sets, in addition to the commonly-used qualitative meta-analysis, to describe, in a comparative manner, the location conditions of the case-studies. We have then assessed the predictive capacity of these conditions using regression analysis. Thus, derived empirical relations are used to create a global map indicating the likelihood of wetland conversion for all wetlands worldwide based on their respective location conditions. 

## Methods

### Meta-analysis

Meta-analysis is a form of systematic review aimed at the statistical evaluation of a large number of case studies. Meta-analysis is especially useful if new (and possibly more structured) data collection is not feasible, due to lack of time and financial resources. In the case of land change, the social and behavioural processes underlying land change patterns in individual case studies can only be studied at the scale of discrete communities or landscapes. Meta-analysis can help to identify commonalities across these case studies and identify which factors (variables) cause individual cases to behave differently [11-19]. A meta-analysis approach, comparable to that of Geist and Lambin [11] for tropical deforestation, has been used here to elucidate the processes driving wetland conversion. Geist and Lambin used 152 sub-national case studies to identify the proximate causes and underlying forces of tropical deforestation. Similarly, case studies of wetland conversion have been collected from peer-reviewed scientific literature collections (‘Web of Science’ and ‘Sciencedirect’), as well as from (non-)governmental research institutes such as Wetlands International (www.wetlands.org) and the US Fish and Wildlife Service. Papers were included if they provided sufficient evidence for wetland conversion, and a description of the proximate causes and underlying forces of the reported conversions. Papers which included only partial information were excluded. The selection procedure and other characteristics of the meta-analysis have been performed and documented according to the Preferred Reporting Items for Systematic Reviews and Meta-Analyses (PRISMA; Checklist S1 and Information S1).

For each of the selected papers, proximate causes and underlying driving forces of wetland conversion have been documented. The descriptions and references to these have been categorized, to allow comparison of the case studies, following the categorization of Geist and Lambin [11]. Where necessary, these categories were adapted and subdivided to accommodate the specific processes indicated in the wetland case studies, and each case study been coded accordingly. Subsequently, a frequency analysis of processes driving wetland conversion was carried out across all case studies. In this analysis both single-factor and multi-factor causations were identified for both proximate causes and underlying drivers of wetland conversion. We also identified the most important interactions between proximate causes and underlying driving forces.

### Meta-regression

To add a more quantitative component to the meta-analysis of proximate causes and underlying driving forces, we carried out an empirical analysis of location factors for the sites of wetland conversion reported in the case studies. All case studies were geo-coded by determining their exact location based on the literature report. As potential determinants of the location of wetland conversion, a number of biophysical and socioeconomic factors were used as independent variables in the regression analyses (Table 1). These factors are selected on the basis of (1) expected relations between the variable and wetland conversion, and, (2) data availability: only maps with global coverage and 5 arcminute resolution are used in the analysis. 

**Table 1:** Explanatory factors used for the wetland conversion regression analysis.

	**Explanatory factor**	**Description**	**Unit**	**Source**
**Biophysical factors**	Temperature	Annual mean (mean of monthly mean).	°C	worldclim.org
	Precipitation	Annual mean (mean of monthly mean).	Mm	worldclim.org
	Slope	Derived from Altitude 30 sec map.	degrees	worldclim.org
	Organic content	Percentage of organic carbon.	% mass	FAO/IIASA/ISRIC/ISSCAS/JRC, [56]
	Histosol	Percentage of histosols.	Ratio (0-1)	FAO/IIASA/ISRIC/ISSCAS/JRC, [56]
	Wetland area	Percentage of wetlands within a 3x3 grid cell area.	Ratio (0-1)	Lehner and Döll, [27]
**Socio-economic factors**	Cropland cover	Average cropland cover within a 3x3 grid cell area.	Ratio (0-1)	Ramankutty et al., [57]
	Agricultural efficiency	Relative measure of land-use intensity.	Ratio (0-1)	Neumann et al., [23]
	Built-up area	Global urban land for c. 2001-2002 based on (MODIS) 500-m satellite data.	% of grid cell.	Schneider et al., [58]
	Population density	Average population density within a 3x3 grid cell area (year 2000).	Nr/km2	CIESIN/CIAT, [59]
	Distance to roads	Distance to nearest road	M	National Geospatial Intelligence Agency (NGA); VMAP0
	Market accessibility	Indicator for the accessibility to markets.	Ratio (0-1)	Verburg et al., [25]
	Market influence	Indicator for market influence.	$/person	Verburg et al., [25]
	Voice and accountability	Captures perceptions of the extent to which a country's citizens are able to participate in selecting their government, as well as freedom of expression, freedom of association, and a free media.	scaled (-2.5 - 2.5)	World Bank, [26]
	Regulatory quality	Captures perceptions of the ability of the government to formulate and implement sound policies and regulations that permit and promote private sector development.	scaled (-2.5 - 2.5)	World Bank, [26]
	Government effectiveness	Captures perceptions of the quality of public services, the quality of the civil service and the degree of its independence from political pressures, the quality of policy formulation and implementation, and the credibility of the government's commitment to such policies.	scaled (-2.5 - 2.5)	World Bank, [26]
	Rule of Law	Captures perceptions of the extent to which agents have confidence in and abide by the rules of society, and in particular the quality of contract enforcement, property rights, the police, and the courts, as well as the likelihood of crime and violence.	scaled (-2.5 - 2.5)	World Bank, [26]

All maps have global coverage and a resolution of 9.25 x 9.25 km in equal area Eckert IV projection (corresponding to 5 arcminutes at the equator).

Six biophysical factors have been selected based on *a priori* expectations of their role in determining locations for wetland conversion (Table 1). Two climatic factors, temperature and precipitation, are thought to influence wetland conversion, since wetlands are often converted for agricultural purposes, with temperature and precipitation being important for crop growth [20]. Wetland conversion to agricultural land is expected to occur predominantly in areas where temperature and precipitation are not limiting plant growth, i.e. where it is not too cold (permafrost zones) or too dry (arid zones; [21,22]). Hence, a positive relation to both temperature and precipitation is expected. We have assumed that agriculture is not curtailed by high temperatures or excess water, and have set no upper bounds. Furthermore, wetland conversion to agricultural land, and also to settlements, is expected to occur on flat terrain or in areas with gentle slopes, conducive to cropland management and to the construction of houses and infrastructure. The organic content of the soil and the occurrence of Histosols (soils that are rich in organic matter) are considered to positively influence wetland conversion for peat harvesting. High levels of organic matter in the soil indicate suitability for peat extraction for fuel or horticulture. Finally, the relation with wetland area can either be positive or negative. It appears to be more efficient (cost-effective) to reclaim a relatively large wetland area in one go, rather than to reclaim many small areas separately. On the other hand, when land is converted by human activities, small patches of wetland in the converted area may easily be lost.

Ten socio-economic factors have been selected (Table 1). The area occupied by cropland adjacent to the wetland is considered to be positively related to wetland conversion. Locations with existing cropland are expected to be more vulnerable to expansion, since it is more efficient to expand cropland than to create new cropland areas on more remote, uncultivated land. In existing cropland areas the necessary facilities like transport routes to markets, housing and labour, are already available. Conversion of wetland to agricultural land requires a relatively high input of human activity (e.g., large-scale drainage, logging) and a positive relation with the technical efficiency of the agricultural production is expected. Technical efficiency is a measure of land-use intensity, with high values for locations where production is close to the maximum potential production, and low values for extensive systems which tend to have low levels of inputs [23]. Built-up area, population density, distance to roads, market accessibility and market influence are also expected to be positively related to wetland conversion. These factors indicate relatively high human pressures on land resources in an area, for example through settlement expansion. Wetlands located close to markets and roads are easily accessible and, hence, more vulnerable to conversion (e.g., 24). The products obtained from converted wetlands (e.g., crop harvest, livestock products, peat) are traded at the markets. Wetlands close to important markets (i.e., markets that are easily accessible and have a high GDP/capita) are expected to be especially vulnerable to conversion, because more capital is available for investments [25]. Wetland conversions may also be stimulated or constrained by government regulations and policies, and for this reason, we have included four World Bank governance indicators: Voice and Accountability, Regulatory Quality, Government Effectiveness and Rule of Law (described in Table 1, [26]).

The value of the dependent variable in the logistic regression analysis is either 0 (no wetland conversion) or 1 (wetland conversion). The case studies obtained from the meta-analysis are used as observations of recent wetland conversion (predominantly in the period 1950-2000; value=1). Since no case studies are available that describe the persistence of wetlands, an alternative approach has been used to create such counterfactual cases (value=0). From the Global Lakes and Wetland database [27], 105 locations were randomly selected (excluding reservoirs and 0-25% wetland complexes) and labelled as ‘no wetland conversion’ sites (value=0). The selection was stratified in 8 world regions (North, Central and South America, Europe, Africa, Russia & China, Middle East & India, and Southeast Asia & Australia), to ensure similar numbers of ‘conversion’ and ‘no conversion’ observations in each region. Four different samples were obtained to investigate the robustness of the findings. 

A logistic regression has been used to estimate the relation between the occurrence of wetland conversion and the independent variables. A Principal Component Analysis (PCA) has been used to examine co-linearity among explanatory factors (Information S2). From each group of variables clustered closely to a principal component, only one variable was selected to minimize the potential impact of co-linearity on the estimated model. The ROC (Receiver Operating Characteristic) was used to assess goodness of fit of the logistic regressions [28]. The ROC relates the fraction of true positives to false positives, and is a frequently used measure of fit in logistic regression. A value of 0.5 indicates the regression model is as good as random; a value of 1 indicates a perfect fit. The resulting regression equations have been used to calculate the probability of wetland conversion of wetlands worldwide using the spatially explicit values of the location factors.

## Results

### Meta-analysis

**Figure 1:**
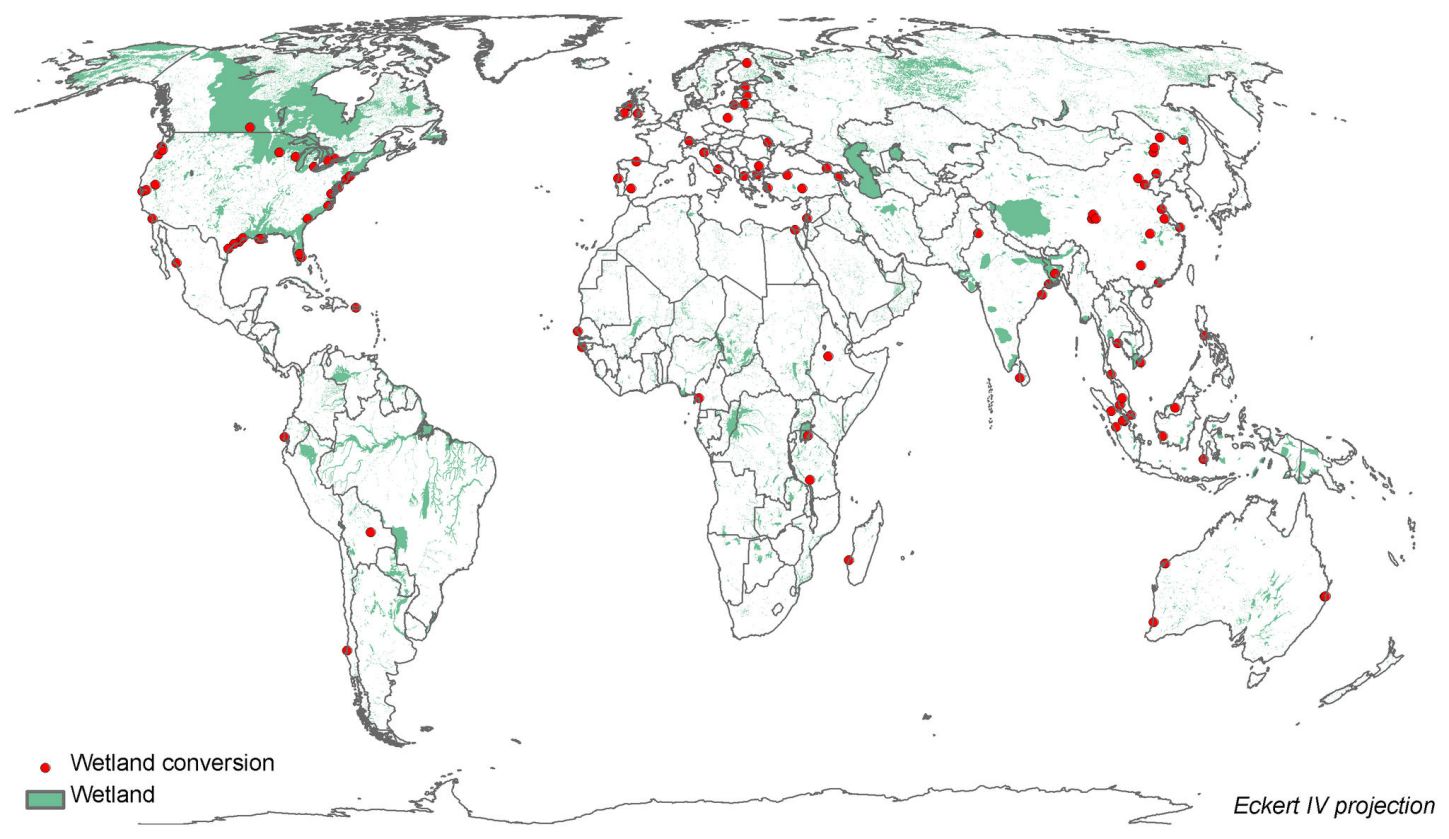
Sites of wetland conversion. In green wetland areas (including lakes and areas with partial wetland cover) from the Global Lakes and Wetland Database (from Lehner and Döll, [27]).

**Figure 2:**
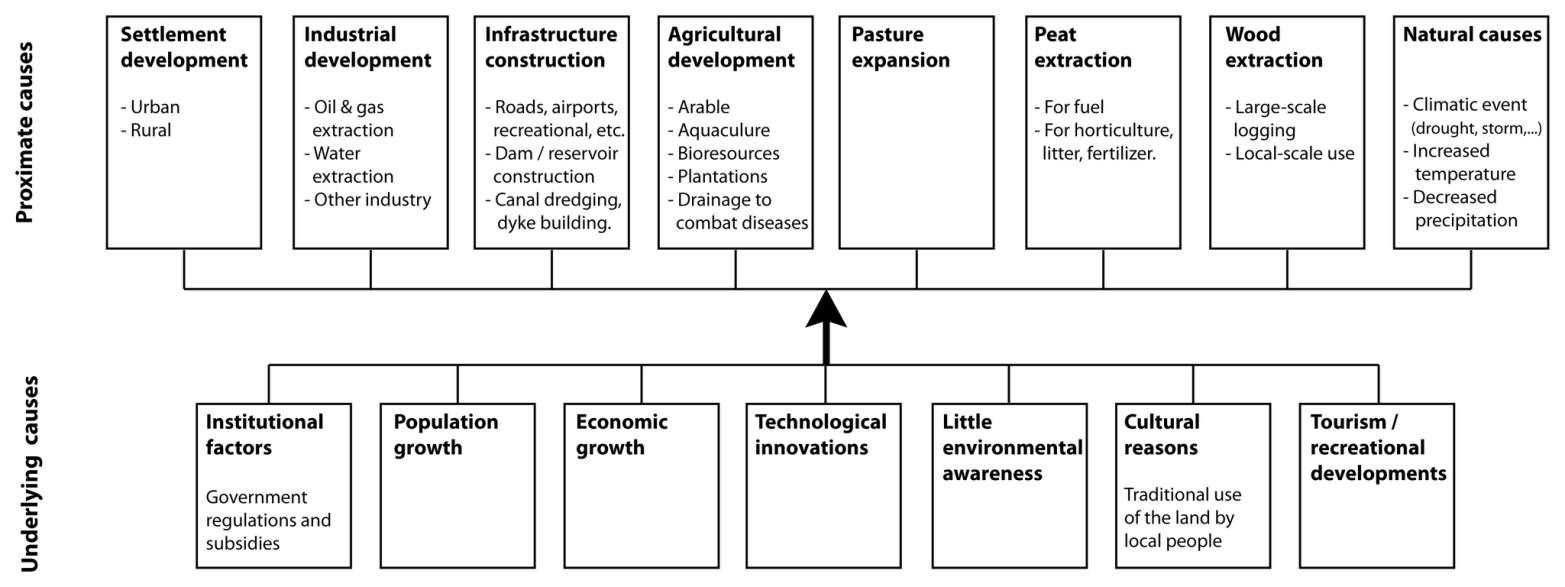
Proximate and underlying drivers of wetland conversion.

In total, 105 case studies were found for which proximate causes and underlying driving forces of wetland conversion could be traced (Figure 1; for a full list and documentation of cases see Information S3). The cases are obtained from 88 papers or reports, with some papers describing wetland conversion at different sites. The collected papers describe wetland conversion from about 1850 onwards, with the period between 1950 and 2000 occurring most frequently. Areas of wetland conversion range from 1 km^2^ to about 150.000 km^2^ (average = 4300 km^2^, median = 97 km^2^). The identified proximate causes and underlying forces of wetland conversion are presented in Figure 2.

### Proximate causes

**Figure 3:**
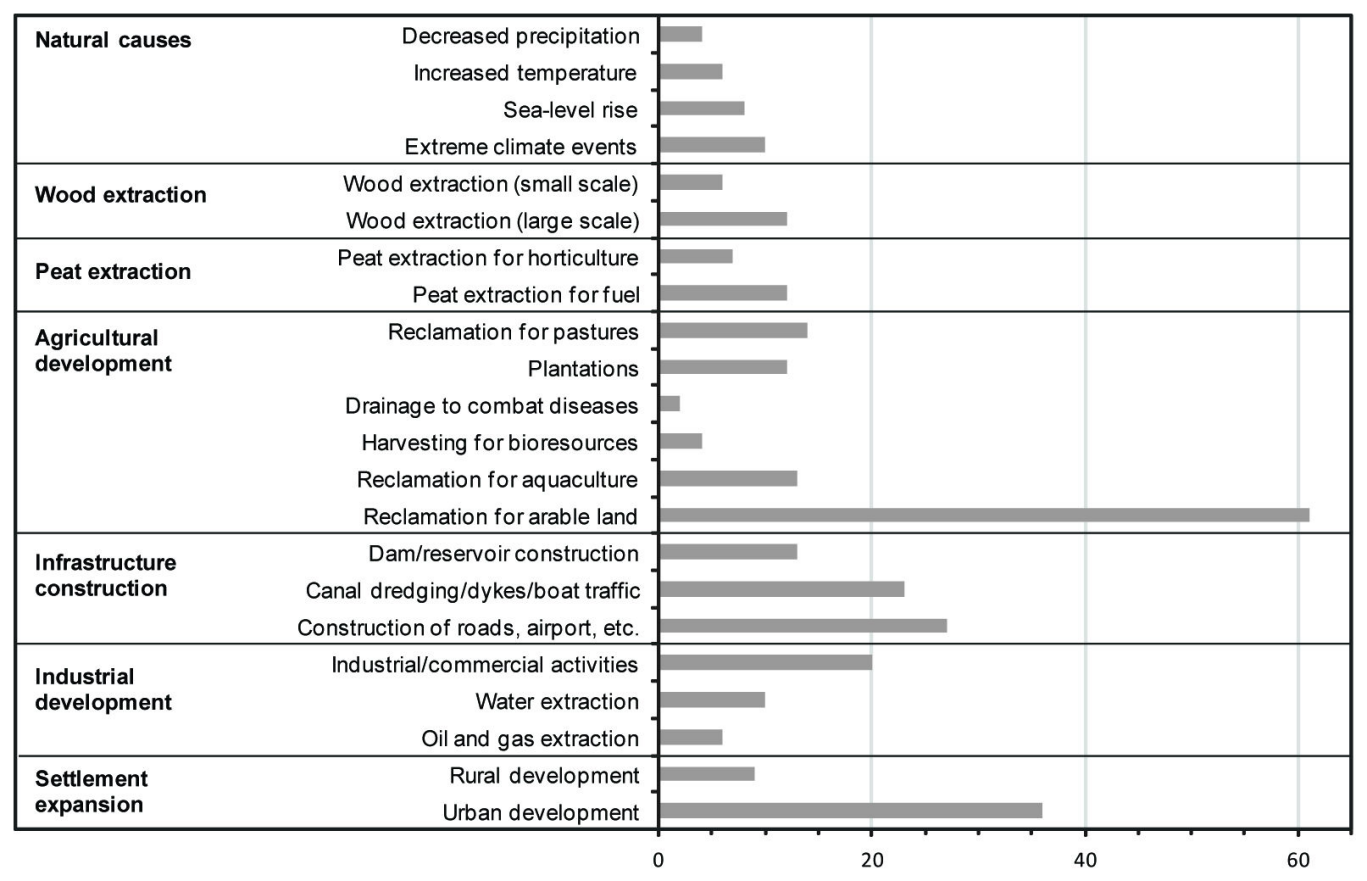
Number of times proximate causes of wetland conversion are documented in the 105 analyzed case-studies.

Expansion of arable land and urban land (sub-categories) are important proximate causes of wetland conversion, documented 61 and 36 times respectively (Figure 3; Information S4). The importance of urban expansion as a cause of wetland conversion can largely be explained by the high population density of many of the world’s largest deltas and floodplains (e.g., 29). Infrastructure construction (e.g., roads, dams, canals, dyke constructions) is another important proximate cause of wetland conversion. Natural causes and low-intensity human activities, such as small-scale wood extraction and harvesting for bio-resources, are less commonly documented as proximate causes of wetland conversion. 

**Figure 4:**
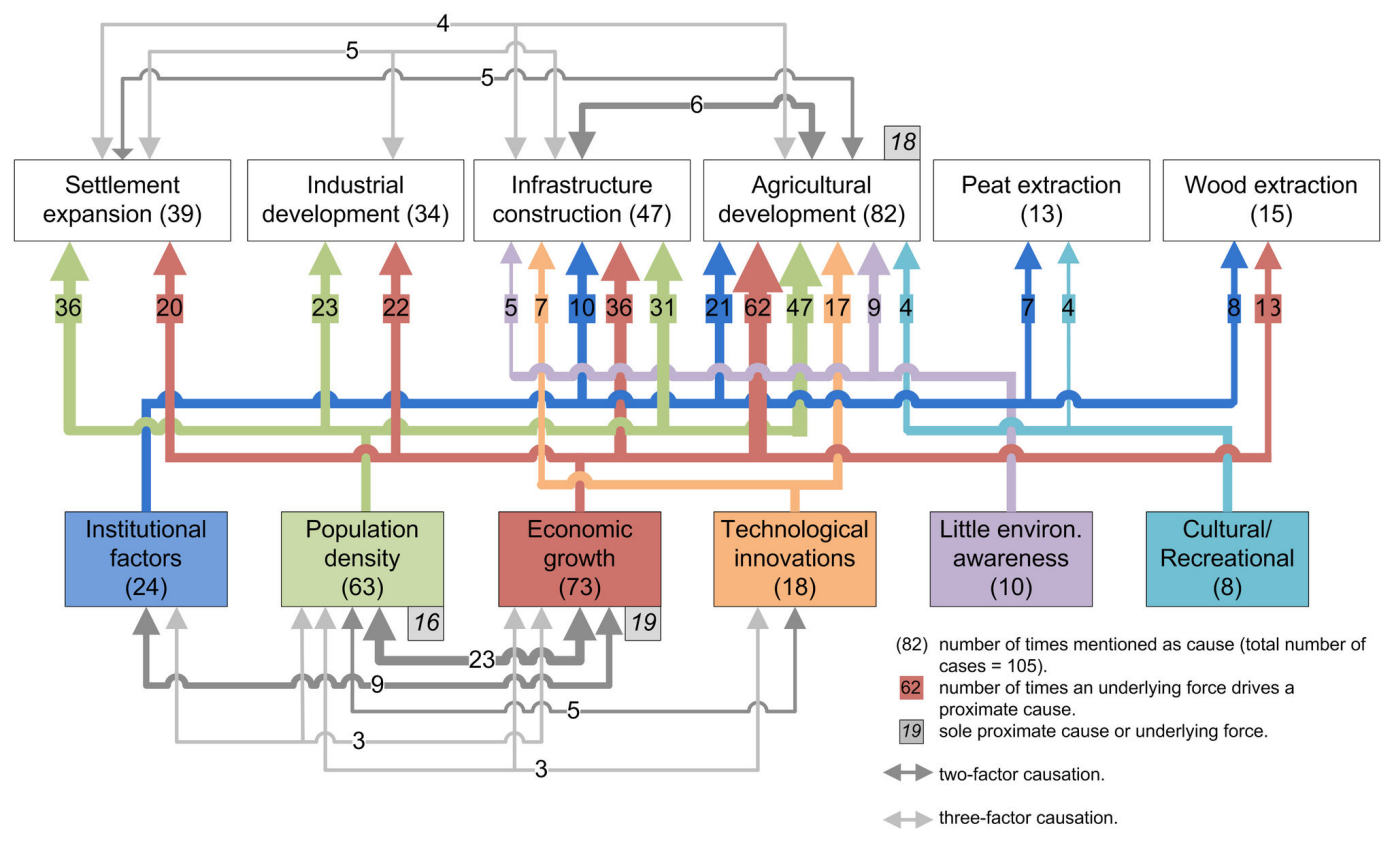
Most frequent occurring combinations of proximate causes and underlying forces of wetland conversion. Agricultural development includes pasture expansion. For each proximate cause at least the two most important underlying forces are indicated, and for each underlying force at least two associated proximate causes indicated.

Many cases mention a combination of proximate causes leading to wetland conversion. Most frequently a two- or three-factor causation is found, although agricultural expansion is reported 18 times in the 105 cases as the sole cause of wetland conversion (Figure 4; Information S5). Most two- and three-factor causations include agricultural expansion, together with any of the other proximate causes. For example, construction of infrastructure (e.g., dykes, roads) is often a requirement in reclaiming wetland for agriculture. Industrial development and settlement expansion is also frequently associated with infrastructure construction. Agricultural development and settlement expansion are often simultaneous, which is indicative of the congruence of expanding settlements and agricultural development. 

### Underlying driving forces

**Figure 5:**
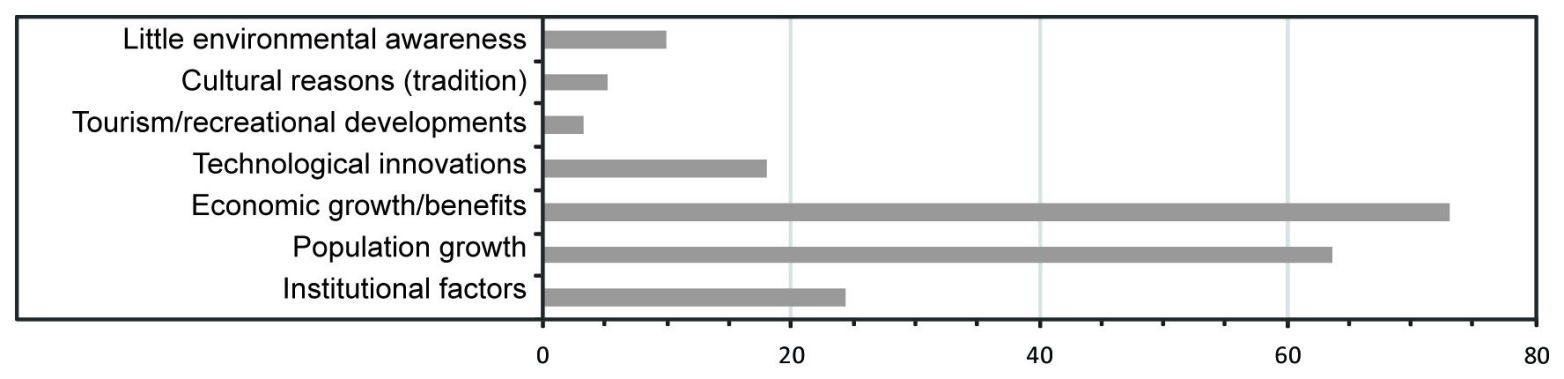
Number of times underlying forces of wetland conversion are documented in the 105 analyzed case-studies.

The most important underlying driving forces of wetland conversion are population growth and economic growth (Figure 5). Underlying driving processes are mostly single- or two-factor causations (Information S6). Population growth and economic growth may occur as a single underlying driving force of wetland conversion (recorded 16 and 19 times respectively), or combined as a two-factor causation (23 times; Figure 4; Information S6). Other important two-factor causations are economic growth and institutional factors (mainly governmental subsidies), and population growth and technological innovation, which are documented 9 and 5 times respectively. Three-factor and more causation occur less frequently.

### Interactions between underlying drivers and proximate causes

Wetland conversions are the results of different combinations of underlying forces and proximate causes. Population density and economic growth are the most important forces driving wetland conversion by agricultural development, infrastructure construction, industrial development and settlement expansion (Figure 4), which frequently occur concurrently (Information S5 and S6). For settlement expansion, population density is a more important underlying force than economic growth: it is mentioned as underlying force in 36 out of 39 settlement expansion cases (=92%), while economic growth is mentioned 20 times (=51%). For agricultural development and infrastructure construction, economic growth is more important than population density: economic growth is mentioned in 76% and 77% of the cases respectively, whereas population density is mentioned in 57% and 66% of them. For industrial development, population and economic growth contribute equally. The most frequently- mentioned underlying forces of wetland conversion by peat extraction are institutional factors and cultural reasons. These interactions have occurred for example in Northern Ireland, where, in the early 1980s, governmental institutions promoted the peat industry by accepting the view of peatland as wasteland that could be exploited to create jobs, and by awarding grants to buy small fuel-extraction machines [30]. In the same country, peat extraction for fuel has long been part of the cultural tradition. Another example comes from the former Soviet Union republics, where, since the 1920s, peat has been a strategic fuel resource and was a main factor in the ambitious project to promote the industrial development of the Soviet Union [31]. In these regions, the use of peat also has a long cultural tradition. Wetland conversion by wood extraction is mainly driven by economic growth and institutional factors. In Finland for example, a national development programme for forestry, with a special focus on drainage of peat soils, was launched in 1964, after which an intensive period of drainage occurred [32]. 

### Meta-regression analysis

After the data reduction analysis by PCA (detailed results reported in Information S2), the following independent variables have been included in the regression model: temperature, precipitation, slope, soil organic content, wetland area, cropland area, built-up area, distance to roads, population density, market influence and regulatory quality. Both forward and backward stepwise selection methods were used to select the independent variables in the regression model using the 4 alternative data sets of ‘no conversion’ cases (Table 2). Slope, precipitation, organic content, distance to roads and regulatory quality were never statistically significant, and have been excluded in subsequent regression analyses. Market influence, wetland area, and mean annual temperature, in this order, were most important and were significant in each of the four cases. Built-up area and cropland area were included in respectively five and four logistic regressions (Table 2). Population density was significant in one regression.

**Table 2:** Independent variables explaining the occurrence of wetland conversion for four different data sets.

	**Data set 1**		**Data set 2**		**Data set 3**		**Data set 4**	
Selection method:	Back	For	Back	For	Back	For	Back	For
Slope								
Temperature	+	+	+	+	+	+	+	+
Precipitation								
Market influence	+	+	+	+	+	+	+	+
Population density							+	
Built-up area	+	+	+		+	+		
Distance to roads								
Wetland area	-	-	-	-	-	-	-	-
Cropland area			+	+	+	+		
Organic content								
Regulatory Quality								
ROC	0.908	0.908	0.884	0.880	0.905	0.905	0.908	0.887

The sign of each significant variable is indicated with ‘+’ and ‘-‘. Back = backward selection method and For = forward selection method (Probability for stepwise selection: P_in_=0.01, P_out_=0.02).

Next, logistic regressions were run again using a single set of input variables for each data set to test the sensitivity of the parameter estimates for the alternative data sets. We selected market influence, wetland area, mean annual temperature and built-up area as most common variables (always or most of the cases significant parameters). Although cropland area was also included 4 times, we did not select this parameter because it highly correlates with built-up area (Information S2). The analysis resulted in four regression equations with the same independent variables and slightly different coefficients: only built-up area varied by a factor 2 between data sets (Table 3). Overall, the coefficients are very similar indicating the robustness of the resulting regression towards alternative selections of non-converted locations. The high values for the ROC statistic also indicate a good fit of the regression models, and these ROC’s are similar to those of the stepwise models presented in Table 2.

**Table 3:** Regression coefficients (and Standard Error: ±) and ROC values for the 4 data sets, using temperature, market access, built-up area and wetland area as independent variables to explain the occurrence of wetland conversion.

	**Constant**	**Temperature**	**Market influence**	**Built-up area**	**Wetland area**	**ROC**
**Data set 1**	0.25±0.35	0.06±0.02	0.39±0.11	1.52±0.73	-3.86±0.64	0.908
**Data set 2**	0.21±0.32	0.05±0.02	0.47±0.11	0.03±0.02	-3.45±0.55	0.861
**Data set 3**	0.41±0.35	0.06±0.02	0.38±0.11	0.14±0.07	-3.76±0.59	0.889
**Data set 4**	0.43±0.36	0.06±0.02	0.44±0.11	0.31±0.15	-3.88±0.60	0.904

To test if our data set can also be used to predict rates of conversion, we carried out a linear regression using the rate of wetland conversion (km^2^/yr) as a dependent variable and the same set of explanatory variables (Table 2) as independent variables. We could derive conversion rates for 85 case studies. While our logistic regression showed that the occurrence of conversion could be predicted quite well, a low R^2^ (0.19) implies that conversion rates are more difficult to predict. Only market influence was statistically significant (p=0.026), with a negative slope. This suggests that, during the time span covered by our data, conversion has occurred at high rates in relatively remote wetland areas (e.g., parts of Canada and the USA, Finland, former Soviet Union republics, Kalimantan). 

**Figure 6:**
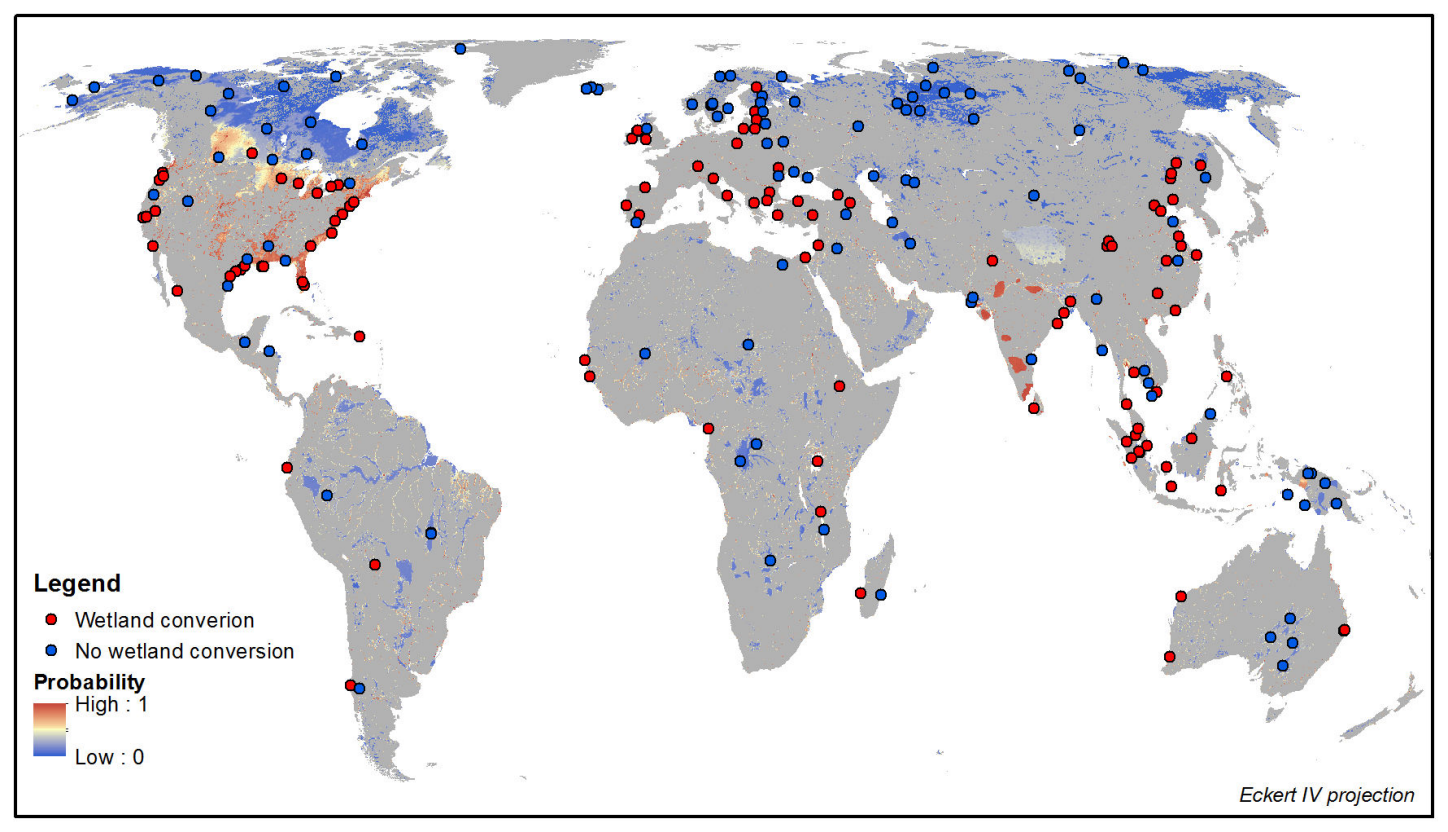
Probability of conversion of wetland areas and converted and non-converted wetland sites (data set 4). Grey areas are non-wetland areas. Wetland areas are defined based on the Global Lakes and Wetland Database [27].

The coefficients of the regression using Data Set 4 have been used to create a global wetland conversion probability map (Figure 6). Although the regression using Data Set 1 resulted in a slightly higher ROC, the coefficient for built-up area is relatively high for this data set (deviates) compared to the other data sets. We therefore did not choose to use Data Set 1, but used Data Set 4 instead. The probability has been calculated for 5 arcminute grid cells that contain more than 10% wetland, as defined by the Global Lakes and Wetlands Database [27]. This map identifies locations where conditions are similar to those where conversion was reported, and which may be interpreted as areas vulnerable to future conversion (assuming that the same factors determine wetland conversion in the future). The probability for wetland conversion is especially high in North America (the USA and southern Canada), Europe, India, China and Indonesia. These areas are often densely populated, with large areas of cropland. The median probability of all converted and non-converted wetland sites (data set 4) is respectively 0.87 and 0.15, which confirms the good fit of the model. 

Sites of wetland conversion usually correspond with a high probability, while sites with no wetland conversion correspond with low probabilities (Figure 6). Sometimes, a non-converted wetland site is found in an area with a high probability of conversion, for example in the south of the USA. This may, for example, be due to the protected status of such locations. Other mismatches between the state of wetland conversion and the predicted probability may be caused by determinants not included in the analyses.

## Discussion and Conclusions

This study applies the method of Geist and Lambin ([11,13]) to study conversion of wetlands at the global scale, making it comparable with meta-studies of other land use changes. A new aspect of our meta-study is the additional quantitative analysis, which has been used to calculate the probability of wetland conversion worldwide allowing an extrapolation of the findings beyond the case studies. The meta-study identifies common driving forces of wetland conversion. The results provide an indication of factors and processes important for land change. All of these are proxies for different causal mechanisms. Such underlying processes can only be revealed in more detailed studies. For example, an identified cause-effect pattern may be caused by different decision patterns and strategies. 

Our study reveals that economic growth and/or population growth are the most important underlying driving forces of one to three-factor proximate causes of wetland conversion that often include agricultural development and/or settlement expansion, associated with, for example, industrial development and infrastructure construction. In the meta-analysis studies of Geist and Lambin ([11,13]) three or more underlying processes drive two to three proximate causes. Hence, we found that, in most cases, less underlying processes drive one to three-factor proximate drivers of wetland conversion. If we presume that factors driving land change are equally well-documented for the different meta-analysis studies, we could speculate that the pathways of wetland conversion are dominated by a limited number of main factors, as opposed to the multi-factor causation found for deforestation and desertification.

Meta-analyses of land change have shown that combinations of multiple and coupled social and biophysical factors drive land change, which may be region specific [11-16]. However, similarities in the factors driving land change may be derived from the various meta-analyses. Important proximate causes of both deforestation and desertification are agricultural activities, infrastructure extension (including settlements) and wood extraction. These are also important proximate causes of wetland conversion, with the exception of wood extraction, which is relatively less important for wetland conversion. In some regions, for example in Finland, wood extraction is the main proximate cause of wetland conversion [33]. Agricultural activities are the leading proximate cause for all three conversion types (deforestation, desertification and wetland conversion). For desertification, increased aridity is also an important proximate cause [13]. Market strength is an important underlying force of wetland conversion (this study), deforestation [11], desertification [13], agricultural intensification in the tropics [14], decrease in swidden cultivation in tropical forest-agriculture frontiers [15] and urban land expansion [16]. These studies also demonstrate that (agricultural) policies, institutional, technological and demographic factors are important underlying forces of land change. There are however also underlying forces that are especially important for a specific land change process. For example, climatic factors (especially a decrease in rainfall) and land tenure arrangements are important underlying forces of desertification [13]. An important factor associated with agricultural intensification in the tropics is property regime [14], which is not mentioned in any of the wetland conversion studies. 

The market influence index is positively related to wetland conversion, indicating that wetlands close to strong (and accessible) markets are especially vulnerable for wetland conversion (Table 3). This appears plausible because economic exploitation of wetlands, and in a broader context global environmental change, is favoured by market demand and access, and the availability of investment capital [25]. As mentioned above, market access and influence has been identified in other meta-analysis studies as a main determinant of land change. Another important process driving wetland conversion is settlement expansion, especially of urban areas, where market accessibility is comparatively high. Hence, this also partly explains the positive relation to market influence. 

Wetland area is negatively related to wetland conversion (Table 3). This implies that, based on our study cases, relatively large wetland areas are less vulnerable for conversion compared to smaller wetland areas. Figure 6 indeed shows that the large wetland areas in for example northern Canada, South America and Siberia have a low probability for wetland conversion. The conversion probability in the large wetland area along the southeast coast of the USA is relatively high however, but, these are wetland complexes (patches of wetland).

The consistent significance of mean annual temperature may reflect the stronger pressure for the more limited land where water is abundantly available at lower latitudes (Table 3). Climate zones of higher temperature are subject to higher evapotranspiration, which may well restrict the land suitable for agriculture more strongly to wetlands than elsewhere at higher latitudes. This is probably reflected in the prevalence of irrigated agriculture [21].

Built-up area is positively related to wetland conversion (Table 3). The principal component analysis showed that built-up area co-varied distinctly with market influence, cropland area and population density (Information S2). Population growth has been mentioned as an underlying force of wetland conversion in 63 out of the 105 case studies (Figure 5), particularly driving wetland conversion by settlement expansion, industrial development, infrastructure construction and agricultural development (Figure 4). Population growth is identified as highly correlated to changes in inundated surface by Prigent et al. [34] and also mentioned as a dominant driver of land change in many other studies (e.g., 11,13-16). It is seen to put pressure on land resources and to stimulate technological and social advances, as an ultimate driver, although its relative importance among drivers of environmental change is still the subject of debate [35,36]. 

We did not include cropland area in the final set of parameters, but it was significant in 4 out of 8 regressions (Table 2). We found a positive relation with cropland area. In the method section, we hypothesized that the reason for this is efficiency. In existing cropland areas the necessary facilities like transport routes to markets, housing and labour, are already available. Case studies from the meta-analysis confirm this hypothesis. For example, in the Prairie Pothole Region in North America, pothole wetlands surrounded by farmland have been drained to create additional cropland [37]. Similar land change patterns of cropland gradually invading wetlands are observed in China, for example in the Sanjiang Plain [38]. In Sri Lanka, marsh loss occurred due to pressures induced by surrounding agricultural land, settlements and industrial land [39]. 

When our logistic regression was restricted to cases after 1980, only the factor built-up land remained significant. This may suggest a possible reflection of gradual historical change. However, this is not supported by a frequency analysis of cases after 1980: besides settlement development, other proximate causes remain equally important. In addition, the most important underlying driving forces are still economic and population growth (Information S7).

In our analysis we used both a case-study meta-analysis and a meta-regression analysis. These approaches complement each other. The case-study analysis provides insight into the relations between different (combinations of) proximate causes and underlying driving forces. However, case studies are not consistent in describing the drivers of land change, and some drivers may not be considered or reported. The meta-regression provides a new method to analyse determinants of land change. For evidence of wetland conversion we currently need to rely on case-study reports. Remote-sensing based observations of wetland conversion mostly address local to regional areas (e.g., 39-44). The meta-regression adds to the case-studies analysis because of its global consistency. Vice versa, the case study meta-analysis adds to the regression, with detailed descriptions of causal relations between driving processes and land change. In this study, the two approaches confirmed the most important drivers of wetland conversion. For example, expansion of arable land is an important proximate cause, which relates to the determinant cropland area. Population density is identified as a key underlying driving force in the case study analysis, and it underlies, or at least covaries, with market influence, cropland and built-up area, which were significant determinants of wetland change in the regression analysis. Similarly, economic growth is often mentioned in the case study papers as an underlying driver force of wetland conversion, while market influence is the significant determinant in our logistic regressions, consistently explaining greatest variability.

Rudel et al. [12] suggest that for meta-analysis of qualitative data the use of a Qualitative Comparative Analysis (QCA) is appropriate. QCA is a technique that groups cases, using Boolean algebra, to create sets of factors, which, in combination, cause a particular condition [45]. There should be both a ‘land change’ and ‘no land change’ condition to make pair-wise comparisons. Land change case studies are strongly biased towards areas with (dramatic) change. The absence of case studies describing drivers of no change means that QCA is not feasible for application in many land change studies. Although the meta-regression approach also requires ‘no conversion’ observations these do not require full case-study descriptions, as use is made of spatial data sets as independent variables. However, the selection of ‘no conversion’ locations introduces uncertainty as the scale of mapping and uncertainty in the global wetland map may cause some of the selected locations to be (partially) converted in reality. The possible influence of the selection procedure on the results of this study was tested by using an alternative approach to select these counterfactual locations. Here, ‘no conversion’ locations were selected from the Protected Planet database (protectedplanet.net), which delimits protected wetland areas, which have certainly persisted during the period 1950-2000. The disadvantage of this approach is that a protected status might imply that these wetlands are or were threatened, confounding the analysis. A test where we have conducted the same analysis using four random selections of locations within protected wetlands indicated that similar factors are significant in the regressions (for more details see Information S8). In particular, the analysis confirms the overriding importance of market influence.

Alternatives to the regression analysis in our study are approaches that are especially equipped to deal with presence-only data, avoiding the use of an artificial set of ‘no change’ locations. Such approaches are common in species distribution modelling, an example of which is MaxEnt [46,47]. The MaxEnt approach relies on an exponential model (equivalent to a Gibbs distribution) to estimate the ratio of the probability density of covariates (indicating location conditions) at presence sites and the probability density of covariates across the study area, for which a random background sample within the area of interest is taken (for details see 48). We have, however, chosen to use randomly-selected counterfactual locations within world regions to achieve a regional balance in the distribution of the counterfactuals with the observations with reported conversion. A random background would put a stronger weight on the larger remaining wetland areas, while smaller wetlands in other world regions would be ignored. 

As globally-consistent observations of wetland conversion are absent, it is difficult to check the reliability of the probability map presented in this paper. The only global scale data on wetland conversion is the global database of land surface water dynamics based on remote sensing images covering the 1993-2007 period [34]. However, these data include all (seasonally) inundated areas such as wetlands, river flood plains and irrigated areas. In many cases, wetland areas are converted into irrigated agriculture or fish ponds so that some locations of conversion may not be detected. A visual comparison of the changes in inundated area extent and our map of wetland conversion probability reveals a large correspondence. The patterns in the USA, and South and South-East Asia, show a particularly strong correspondence. Other locations, such as along the Amazon River and in Southern China, reveal more differences, which may be attributed to other processes (e.g., changes in flooding regime of rivers), or expansion of irrigated areas or local processes not captured by the meta-regression. The comparison shows, however, the prospect of refined observation techniques, in combination with meta-analysis of case studies, to inventorise and understand wetland conversion at a global scale.

Inherent to meta-study analyses, the results depend on the completeness and accuracy of the case study descriptions. A general bias is the question as to whether all drivers of a conversion are described in the (scientific) literature or not. Drivers that are not mentioned in the case study papers may still have had an influence on wetland conversion, while authors may focus on another process. Additional bias may be caused by a non-representative geographical spread of the case studies. For example, in our meta-analysis relatively large numbers of case studies are located in Europe, China, Indonesia and the USA. This may be caused by large-scale research projects supported by (non-)governmental organizations that are available online, such as the report on peatlands in Central and Eastern Europe (by Wetlands International, [49]) and the report on wetlands of the United States (by the U.S. Fish and Wildlife service, [50]). Moreover, other regions may receive extra attention because wetland conversion is associated with other important issues of global significance, such as large-scale deforestation (of amongst others peat swamp forests) in Indonesia. 

Apart from possible bias in the case-study locations and the selected ‘no change’ locations, bias in the regression analysis is mainly introduced by the validity and resolution of the explanatory variable maps. All maps, with the exception of the governance maps, are derived from (the latest version of) 5 arcminute or higher resolution maps (for details see references in Table 1). The governance maps present data at (sub)national level. However, in some countries governance systems can also strongly vary within the national boundaries. 

Case studies provide useful descriptions of the local context and provide depth in the descriptions of processes and underlying drivers of land change. However, case study results are only useful for larger-scale analysis when their results can be generalized. Meta-analysis attempts to achieve this. However, given the qualitative nature, the outcomes are difficult to use in more quantitative approaches for assessment of changes in land use, biodiversity and ecosystem services. The meta-regression approach presented in this paper adds a quantitative component to the analysis by identifying sites with similar location characteristics to those which have been converted in the past. Such maps can help to inform the construction of integrated assessment models that use probability maps to identify locations of potential conversion. The regression equations from our study can, e.g., be implemented in the IMAGE-GLOBIO model chain [51,52] and be used to predict the probability of wetland conversion, and therewith loss of biodiversity. While the probability maps provide an overview of locations with similar location conditions as those that were converted in the recent past, they do not provide insight into the complexity of proximate causes and underlying driving processes of wetland conversion. This insight is provided by the traditional meta-analysis that may help the assessment of wetland conversions by indicating the proximate causes that may be related to frequently modelled processes in global models [53,54]. In this sense, the meta-analysis performed in this paper helps to better empirically ground global models and helps translate the rich knowledge attained in local studies to global scale assessments. Such empirical grounding of global assessment models in the reality of complex systems processes will help to make the models more realistic and useful for decision making [55]. 

## Supporting Information

Checklist S1 **PRISMA 2009 Checklist.**
(DOCX)

Information S1 **PRISMA 2009 Flow Diagram.**
(DOC)

Information S2 **Principal Components Analysis results.**
(DOCX)

Information S3 **Case-study documentation.**
(XLSX)

Information S4 **Proximate causes and underlying driving forces of wetland conversion and the number of times each processes is mentioned in the case study papers (N=105).**
(DOCX)

Information S5 **Absolute number, relative and cumulative contributions of combinations of proximate causes (expressed in single or multiple-factor causations) of wetland conversion.**
(DOCX)

Information S6 **Absolute number, relative and cumulative contributions of combinations of underlying forces (expressed in single or multiple-factor causations) of wetland conversion.**
(DOCX)

Information S7 **Results of a frequency analysis including only the cases since 1980.**
(DOCX)

Information S8 Logistic regression using counterfactual locations based on the Protected Planet database.
(DOCX)
