# Supplementary material for: Drivers of Wetland Conversion: a Global Meta-Analysis
Source: PLoS One. 2013 Nov 25;8(11):e81292. doi: 10.1371/journal.pone.0081292 (PMC3840019; doi:10.1371/journal.pone.0081292)
Supplement: Checklist S1 — PRISMA 2009 Checklist. (DOCX) [file pone.0081292.s001.docx]

| **Section/topic** | **#** | **Checklist item** | **Reported on page #** |
| --- | --- | --- | --- |
| **TITLE** | | |  |
| Title | 1 | Drivers of Wetland Conversion: a Global Meta-analysis | Title page |
| **ABSTRACT** | | |  |
| Structured summary | 2 | Meta-analysis of case studies has become an important tool for synthesizing case study findings in land change. Meta-analyses of deforestation, urbanization, desertification and change in shifting cultivation systems have been published. This present study adds to this literature, with an analysis of the proximate causes and underlying forces of wetland conversion at a global scale using two complementary approaches of systematic review. Firstly, a meta-analysis of 105 case-study papers describing wetland conversion was performed, showing that different combinations of multiple-factor proximate causes, and underlying forces, drive wetland conversion. Agricultural development has been the main proximate cause of wetland conversion, and economic growth and population density are the most frequently identified underlying forces. Secondly, to add a more quantitative component to the study, a logistic meta-regression analysis was performed to estimate the likelihood of wetland conversion worldwide, using globally-consistent biophysical and socioeconomic location factor maps. Significant factors explaining wetland conversion, in order of importance, are market influence, proximity of other wetlands (lower conversion probability), mean annual temperature and adjacent cropland or built-up land. In a regression analysis of recent cases (after 1980), only adjacent built-up land remains as a significant factor. A frequency analysis including only cases after 1980, however, demonstrated that, compared to the analysis including all cases, in recent years still the same proximate causes and underlying forces are important drivers of wetland conversion. These results support the outcomes of the meta-analysis of the processes of conversion mentioned in the individual case studies. In other meta-analyses of land change, similar factors (e.g., agricultural development, population growth, market/economic factors) are also identified as important causes of various types of land change (e.g., deforestation, desertification). Meta-analysis helps to identify commonalities across the various local case studies and identify which factors (variables) cause individual cases to behave differently. The meta-regression provides maps indicating the likelihood of wetland conversion worldwide based on the location factors that have determined historic conversions. | Abstract |
| **INTRODUCTION** | | |  |
| Rationale | 3 | Studies reporting wetland conversion are mostly conducted for single locations. A more comprehensive understanding of which are the most important drivers of wetland conversion at a global scale, and how these interact, is currently lacking, in spite of the importance and scale of wetland conversion. Processes of wetland loss and degradation reduce the capacity of wetlands to provide valuable ecosystem services to humanity like water supply, flood control, carbon storage, maintenance of biodiversity, retention of sediment and nutrients, and recreation. Such services have both global significance and local value, and their conservation value is argued for widely. Meta-analyses have already been done for tropical deforestation, desertification, agricultural intensification in the tropics, swidden agriculture changes in tropical forest-agriculture frontiers, and for urban land conversions, but not yet for wetland conversion. | Introduction |
| Objectives | 4 | The main objective is to study the most important drivers of wetland conversion at a global scale, and how these interact (the PICOS approach does not apply to this study). | Introduction |
| **METHODS** | | |  |
| Protocol and registration | 5 | Case studies of wetland conversion have been collected from peer-reviewed scientific literature collections (‘Web of Science’ and ‘Sciencedirect’), as well as from (non-)governmental research institutes such as Wetlands International (www.wetlands.org) and the US Fish and Wildlife Service. For each of these papers, proximate causes and underlying driving forces of wetland conversion have been documented. The descriptions and reference to proximate causes and underlying driving forces have been categorized in a number of categories to allow comparison of the case studies. Starting point for this categorization are the categories distinguished by Geist and Lambin [2002: BioScience 52: 143-150]. But, where needed these were adapted and subdivided to accommodate the important differences in processes indicated in the wetland case studies. The identified categories have been used to code each case study and conduct a frequency analysis of processes driving wetland conversion across all case studies. In this analysis both single-factor and multi-factor causations are identified for both proximate causes and underlying drivers of wetland conversion. We also identified the most important interactions between proximate causes and underlying driving forces. | Methods section |
| Eligibility criteria | 6 | The collected case-study papers describe wetland conversion during the period from about 1850 onwards, with the period between 1950 and 2000 occurring most frequently. Areas of wetland conversion range from 1 km2 to about 150.000 km2 (average = 4300 km2, median = 97 km2). Case-studies have been collected from peer-reviewed scientific English literature collections. | Methods & results + Sup. Inf. S4 |
| Information sources | 7 | Case studies have been collected from peer-reviewed scientific literature collections (‘Web of Science’ and ‘Sciencedirect’), as well as from (non-)governmental research institutes (Wetlands International (www.wetlands.org) and the US Fish and Wildlife Service). | Methods section |
| Search | 8 | This has been described at item 5. | Methods section |
| Study selection | 9 | A case-study paper describing wetland conversion has been included in the meta-analysis if both proximate causes and underlying driving factors could be identified. | Methods section |
| Data collection process | 10 | Each case-study paper has been screened by the first author who identified and coded proximate causes and underlying driving factors of wetland conversion. | Methods section |
| Data items | 11 | The following variables have been extracted from each case-study paper: country, region/place, latitude, longitude, wetland type (according to the Global Lakes and Wetland Database), period of wetland conversion, area of wetland that has been converted, reference, codes for proximate causes and underlying driving factors. | Sup. Info S4 |
| Risk of bias in individual studies | 12 | The results of the meta-analysis depend on the completeness and accuracy of the case study descriptions. A general bias is the mere question whether all drivers of a conversion are described in the (scientific) literature or not. Drivers that are not mentioned in the case-study papers may have had an influence on wetland conversion, or otherwise, authors may focus on one particular process. We estimate that the bias introduced by these issues is relatively small, because it is plausible to assume that at least the most important drivers of wetland conversion are always mentioned. Additional bias may be caused by a non-representative spread of the case-studies as has been reported in other meta-analysis. A quantified analysis of bias was not possible, instead, the discussion section identifies and shortly discusses these biases | Discussion section |
| Summary measures | 13 | Output of the frequency analysis: absolute number, relative and cumulative contributions of combinations (expressed in single or multiple-factor causations) of proximate causes and underlying driving forces of wetland conversion. | Methods section |
| Synthesis of results | 14 | The results of the frequency analysis are used to identify both single-factor and multi-factor causations of both proximate causes and underlying drivers of wetland conversion. We also identified the most important interactions between proximate causes and underlying driving forces. | Methods section |

Page 1 of 2

| **Section/topic** | **#** | **Checklist item** | **Reported on page #** |
| --- | --- | --- | --- |
| Risk of bias across studies | 15 | Several case-studies described by the same author(s) as well as geographic spread in case study locations may introduce bias. The risk of geographic bias has been described in the discussion section. | Discussion |
| Additional analyses | 16 | To add a more quantitative component to the meta-analysis of proximate causes and underlying driving forces we carried out an empirical analysis of location factors associated with the locations of wetland conversion reported in the case studies. For that purpose all case studies were geo-coded by determining their exact location based on the literature report. As potential determinants of the location of wetland conversion a set of biophysical and socioeconomic factors has been used as independent variables in the logistic regression analyses. These factors are selected based on (1) expected relations between the variable and wetland conversion, and, (2) data availability: only maps with global coverage and 5 arcminute resolution are used in the analysis.  As a robustness check the regression analysis was repeated for recent cases (after 1980).  A Principal Component Analysis (PCA) has been used to examine co-linearity among explanatory factors. From each group of variables clustered closely to a principal component only one variable was selected to minimize the potential impact of co-linearity on the estimated model. | Methods and results section |
| **RESULTS** | | |  |
| Study selection | 17 | In total 105 case-studies have been included, which were obtained from 88 papers/reports. | Results |
| Study characteristics | 18 | The variables extracted from each case-study paper (item 11) are presented in Table 1 (including citations). | Table 1 |
| Risk of bias within studies | 19 | No quantitative analysis was done on the bias, the discussion section discusses qualitatively the risks of bias | Discussion section |
| Results of individual studies | 20 | Table S1 presents the coding of the individual studies. | Sup. Info. S4 |
| Synthesis of results | 21 | The main results of the meta (frequency) analysis are presented in Supporting Information S5, S6 and S7. The robustness check confirmed the main results. | Sup. Info. S5, S6, S7 |
| Risk of bias across studies | 22 | No quantitative analysis of bias across studies was made. A qualitative description of possible biases was made in the discussion section | Discussion section |
| Additional analysis | 23 | Results of the PCA analysis show that the various indicators are highly correlated. In these cases only one indicator was used for the regression analysis. Correlated variables are: (1) Rule of law, Government effectiveness, Regulatory quality and Voice and accountability, (2) market influence and market accessibility, (3) occurrence of Histosols and soil organic content, and (4) cropland area and the technical efficiency of agricultural production. Of these four groups Regulatory quality, market influence, soil organic content and cropland area were respectively used in the regression analysis.  Regression results show that slope, precipitation, organic content, distance to roads and Regulatory Quality were never statistically significant. Market influence, temperature, wetland area, built-up and cropland area were statistically significant in most/all analyses.  The regression coefficients of the equation with the best fit (high ROC in combination with average coefficient values) have been used to create a global wetland conversion probability map, using market influence, mean annual temperature, built-up area and wetland area as explanatory factors. | Results section |
| **DISCUSSION** | | |  |
| Summary of evidence | 24 | Economic growth and/or population growth are the most important underlying driving forces of one to three-factor proximate causes of wetland conversion that often include agricultural development and/or settlement expansion, associated with for example industrial development and infrastructure construction. The two approaches (meta-analysis and meta-regression) confirmed the most important drivers of wetland conversion. For example, expansion of arable land (proximate cause) relates to cropland area (explanatory factor), economic growth (important underlying factor) relates to market influence (significant explanatory factor), and population density is both an important underlying driving factor and a significant explanatory factor (it covaries with market influence, built-up area and cropland area). The high ROC (0.9) indicates a good fit of the regression model. The meta-analysis helps to identify commonalities across the various local case studies and identify which factors (variables) cause different cases to behave differently. The meta-regression provides maps indicating the likelihood of wetland conversion worldwide, which can be used as input for global scale environmental change assessments. | Results and discussion sections |
| Limitations | 25 | Limitations of this study relate to factors explained at item 15. Furthermore, bias in the regression analysis is mainly introduced by the validity and resolution of the explanatory variable maps. All maps except the governance maps are derived from (the latest version of) 5 arcminute or higher resolution maps (for details see references in Table 1). The governance maps present data at (sub)national level. However, in some countries governance systems can also strongly vary within the national boundaries.  For the regression analysis sites where no wetland conversion has occurred had to be selected. This was done based on the Global Lakes and Wetlands Database. We selected random locations from the global wetland map, assuming that the locations indicated on the map are currently not converted. However, the scale of mapping and uncertainty in the map may result in locations being selected that, in reality, have seen some conversion. This risk is assessed to be small since ‘no conversion’ sites are located in remote areas like northern Canada, northern Europe, Siberia, central Australia and central Africa where the effect of e.g., economic growth and population density is small. In addition, we carried out the same regression analysis but then selecting counterfactual locations randomly from the Protected Planet database. This gave similar regression results. | Discussion section |
| Conclusions | 26 | The main outcomes are described at item 24. The meta-analysis provides insight into the relations between different (combinations of) proximate causes and underlying driving forces, and wetland conversion. The meta-regression approach adds a quantitative component to the analysis that can help to inform the construction of integrated assessment models. The meta-analysis performed in this paper helps to better empirically ground global models and helps translate the rich knowledge attained in local studies to global scale assessments. Such empirical grounding of global assessment models in the reality of complex systems processes will help to make the models more realistic and useful for decision making. | Discussion and conclusion section |
| **FUNDING** | | |  |
| Funding | 27 | This research is funded by the Netherlands Organization for Scientific Research (NWO; project IGLO-830.10.003) and supported by the Netherlands Environmental Assessment Agency. The research contributes to the Global Land Project (<http://www.globallandproject.org>). | Acknowledgements |

*From:*  Moher D, Liberati A, Tetzlaff J, Altman DG, The PRISMA Group (2009). Preferred Reporting Items for Systematic Reviews and Meta-Analyses: The PRISMA Statement. PLoS Med 6(6): e1000097. doi:10.1371/journal.pmed1000097

For more information, visit: **www.prisma-statement.org**.

Page 2 of 2
