# Supplementary material for: Drivers of Wetland Conversion: a Global Meta-Analysis
Source: PLoS One. 2013 Nov 25;8(11):e81292. doi: 10.1371/journal.pone.0081292 (PMC3840019; doi:10.1371/journal.pone.0081292)
Supplement: Information S1 — PRISMA 2009 Flow Diagram. (DOC) [file pone.0081292.s002.doc]

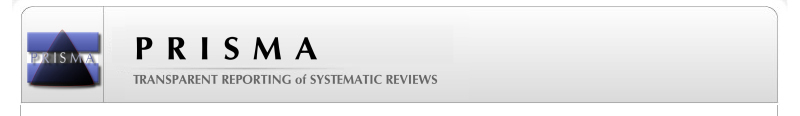
**Supporting Information S2: PRISMA 2009 Flow Diagram**

**Screening**

**Included**

**Eligibility**

**Identification**

Records identified through database searching
(n = 200 )

Additional records identified through other sources
(n = 0 )

Records after duplicates removed
(n = 200 )

Records screened
(n = 200 )

Records excluded
(n = 70 )

Full-text articles assessed for eligibility
(n = 130 )

Full-text articles excluded, with reasons
(n = 25 )

Studies included in qualitative synthesis
(n = 105 )

Studies included in quantitative synthesis (meta-analysis)
(n = 105 )

The numbers in red are estimates. Case-study papers and report were collected by an internet search (Web of Science, Sciencedirect, etc.) and were directly excluded (not documented) if they were not useful for the meta-analyses.
