# Supplementary material for: Drivers of Wetland Conversion: a Global Meta-Analysis
Source: PLoS One. 2013 Nov 25;8(11):e81292. doi: 10.1371/journal.pone.0081292 (PMC3840019; doi:10.1371/journal.pone.0081292)
Supplement: Information S2 — Principal Components Analysis results. (DOCX) [file pone.0081292.s003.docx]

**Supporting Information S3 –PCA analysis**

Results of the PCA analysis show that the four governance indicators are highly correlated (Table S1). Therefore, only one governance indicator, Regulatory Quality, has been used for the regression analysis.

Market influence and market access are also highly correlated (Table S1). Market influence is deduced from a market accessibility index and the Purchasing Power Parity (PPP) per [25]. We chose to include market influence in the regression analysis. The economic status, which is an important underlying driver of wetland conversion (Figure 4), is in this way also (besides market accessibility) indirectly included in the analysis.

Third, the occurrence of Histosols and the soil organic content are highly correlated (Table S1). This is trivial because Histosols are defined as soils that are rich in organic matter [32]. Because organic content is the main variable used to distinguish Histosols, only organic content has been used in the regression analysis.

Finally, cropland area is highly correlated with the technical efficiency of agricultural production at these locations. This means that in wetland locations with large cropland areas the intensity of cultivation is also high. We chose to use cropland area as an independent variable in the regression analysis.

**Table S1.** Principal components loadings. All wetland conversion cases and the no wetland conversion sites are included in the PCA analysis (N=210). The percentage of variance explained by each component is indicated between brackets.

|  | **Principal Component** | | | |
| --- | --- | --- | --- | --- |
|  | **1 (25%)** | **2 (18%)** | **3 (12%)** | **4 (10%)** |
| Distance to roads | .016 | -.509 | .115 | .102 |
| Wetland area | -.136 | -.246 | .312 | .170 |
| Rule of law | .960 | -.121 | .037 | -.085 |
| Government effectiveness | .946 | -.144 | .099 | -.085 |
| Regulatory quality | .963 | -.102 | .089 | -.044 |
| Voice and accountability | .908 | -.231 | .084 | -.056 |
| Percentage of Histosols | -.079 | -.321 | .821 | -.044 |
| Slope | .112 | .029 | -.130 | .162 |
| Market accessibility | .377 | .757 | .087 | .211 |
| Market influence | .540 | .681 | .089 | -.073 |
| Built-up area | .259 | .452 | .028 | .611 |
| Population density | .016 | .411 | .021 | .489 |
| Cropland area | -.133 | .611 | .169 | -.589 |
| Efficiency of agricultural production | -.098 | .593 | .213 | -.623 |
| Soil organic content | -.035 | -.297 | .834 | -.059 |
| Precipitation | -.133 | .224 | .544 | .283 |
| Temperature | -.254 | .436 | .454 | .222 |
