# Supplementary material for: Drivers of Wetland Conversion: a Global Meta-Analysis
Source: PLoS One. 2013 Nov 25;8(11):e81292. doi: 10.1371/journal.pone.0081292 (PMC3840019; doi:10.1371/journal.pone.0081292)
Supplement: Information S4 — Proximate causes and underlying driving forces of wetland conversion and the number of times each processes is mentioned in the case study papers (N=105). (DOCX) [file pone.0081292.s005.docx]

**Supporting Information S5.** Proximate causes and underlying driving forces of wetland conversion and the number of times each processes is mentioned in the case study papers (N=105).

| **Proximate causes** | | | |
| --- | --- | --- | --- |
| Agricultural | 79 | Arable land | 61 |
| development |  | Aquaculture | 13 |
|  |  | Harvesting for bioresources (cranberries, reed cutting, medicinal plants) | 4 |
|  |  | Plantations | 12 |
|  |  | Drainage to combat malaria, dysentery, cholera | 2 |
| Wood extraction | 15 | Large-scale logging | 12 |
|  |  | Local use of wood | 6 |
| Pasture expansion | 14 | - | 14 |
| Settlement | 39 | Urban | 36 |
| development |  | Rural | 9 |
| Industrial/commercial | 34 | Oil and gas extraction | 6 |
| development |  | Water extraction (for e.g. irrigation, hydroelectical power) | 10 |
|  |  | Industrial/commercial activities/extraction: other | 20 |
| Peat extraction | 13 | For fuel (local use versus large-scale peat extraction) | 12 |
|  |  | For horticulture/litter/fertilizer | 7 |
| Infrastructure | 47 | Roads, airport, recreational functions, etc. | 27 |
| construction |  | Dam construction / Reservoirs (sediment deficit) | 13 |
|  |  | Canal dredging/filling/dykes (for land drainage) / boat traffic | 23 |
| Natural causes | 24 | Climatic events: droughts, fires, storms, cyclones | 10 |
|  |  | Increased temperature | 6 |
|  |  | Decreased precipitation | 4 |
|  |  | Sea-level rise | 8 |
| **Underlying factors** | | | |
| Institutional factors (mainly government regulations and subsidies) | | | 24 |
| Population growth | | | 63 |
| Economic growth / benefits | | | 73 |
| Little environmental awareness | | | 10 |
| Technological innovations | | | 18 |
| Cultural reasons (traditional use of the land by local people) | | | 5 |
| Tourism/recreational developments | | | 3 |
