# Supplementary material for: Drivers of Wetland Conversion: a Global Meta-Analysis
Source: PLoS One. 2013 Nov 25;8(11):e81292. doi: 10.1371/journal.pone.0081292 (PMC3840019; doi:10.1371/journal.pone.0081292)
Supplement: Information S5 — Absolute number, relative and cumulative contributions of combinations of proximate causes (expressed in single or multiple-factor causations) of wetland conversion. (DOCX) [file pone.0081292.s006.docx]

**Supporting Information S6.** Absolute number, relative and cumulative contributions of combinations of proximate causes (expressed in single or multiple-factor causations) of wetland conversion (agr = agricultural development, wood = wood extraction, pas = pasture expansion, set = settlement expansion, ind = industrial/commercial development, peat = peat extraction, inf = infrastructure construction, nat = natural causes).

|  | **Absolute number (N=105)** | **Relative contribution (%)** | **Cumulative contribution (%)** |
| --- | --- | --- | --- |
| **Single-factor causation** | **23** | **22** |  |
| agr | 18 | 17 | 17 |
| peat | 2 | 2 | 19 |
| set | 2 | 2 | 21 |
| nat | 1 | 1 | 22 |
|  |  |  |  |
| **Two-factor causation** | **29** | **28** |  |
| agr-inf | 6 | 6 | 28 |
| agr-set | 5 | 5 | 32 |
| agr-nat | 3 | 3 | 35 |
| agr-pas | 3 | 3 | 38 |
| agr-wood | 3 | 3 | 41 |
| agr-ind | 2 | 2 | 43 |
| set-inf | 1 | 1 | 44 |
| set-ind | 2 | 2 | 46 |
| ind-inf | 2 | 2 | 48 |
| ind-nat | 1 | 1 | 49 |
| wood-nat | 1 | 1 | 50 |
|  |  |  |  |
| **Three-factor causation** | **35** | **33** |  |
| agr-set-inf | 4 | 4 | 53 |
| agr-set-nat | 3 | 3 | 56 |
| agr-set-ind | 2 | 2 | 58 |
| agr-ind-inf | 3 | 3 | 61 |
| agr-wood-peat | 3 | 3 | 64 |
| agr-wood-set | 2 | 2 | 66 |
| agr-wood-ind | 1 | 1 | 67 |
| agr-wood-pas | 1 | 1 | 68 |
| agr-pas-inf | 2 | 2 | 70 |
| agr-pas-set | 1 | 1 | 70 |
| agr-infr-nat | 1 | 1 | 71 |
| set-ind-inf | 5 | 5 | 76 |
| ind-inf-nat | 4 | 4 | 80 |
| set-inf-nat | 1 | 1 | 81 |
| pas-inf-nat | 1 | 1 | 82 |
| pas-peat-nat | 1 | 1 | 83 |
|  |  |  |  |
| **Four-factor causation** | **11** | **10** |  |
| agr-set-ind-inf | 3 | 3 | 86 |
| agr-wood-peat-inf | 2 | 2 | 88 |
| agr-ind-peat-inf | 1 | 1 | 89 |
| agr-pas-ind-inf | 1 | 1 | 90 |
| agr-pas-set-ind | 1 | 1 | 90 |
| agr-wood-set-inf | 1 | 1 | 91 |
| set-ind-inf-nat | 1 | 1 | 92 |
| pas-set-inf-nat | 1 | 1 | 93 |
| **Five-factor causation** | **7** | **7** |  |
| agr-set-ind-inf-nat | 3 | 3 | 96 |
| agr-pas-peat-inf-nat | 2 | 2 | 98 |
| agr-set-ind-peat-inf | 1 | 1 | 99 |
| agr-wood-ind-peat-inf | 1 | 1 | 100 |
|  |  |  |  |
| **Total** | **105** | **100** |  |
