# Supplementary material for: Drivers of Wetland Conversion: a Global Meta-Analysis
Source: PLoS One. 2013 Nov 25;8(11):e81292. doi: 10.1371/journal.pone.0081292 (PMC3840019; doi:10.1371/journal.pone.0081292)
Supplement: Information S6 — Absolute number, relative and cumulative contributions of combinations of underlying forces (expressed in single or multiple-factor causations) of wetland conversion. (DOCX) [file pone.0081292.s007.docx]

**Supporting Information S7.** Absolute number, relative and cumulative contributions of combinations of underlying forces (expressed in single or multiple-factor causations) of wetland conversion (pop = population growth, eco = economic growth, ins = institutional factors, tech = technological innovations, cult = cultural reasons, awar = environmental awareness).

|  | **Absolute number (N=105)** | **Relative contribution (%)** | **Cumulative contribution (%)** |
| --- | --- | --- | --- |
| **Single-factor causation** |  |  |  |
| Population growth | 16 | 15 | 15 |
| Economic growth | 19 | 18 | 33 |
| Institutional factors | 2 | 2 | 35 |
|  |  |  |  |
| **Two-factor causation** |  |  |  |
| pop-eco | 23 | 22 | 57 |
| pop-cult | 3 | 3 | 60 |
| pop-tech | 5 | 5 | 65 |
| pop-awar | 2 | 2 | 67 |
| pop-tour | 1 | 1 | 68 |
| eco-tech | 3 | 3 | 70 |
| eco-awar | 2 | 2 | 72 |
| ins-tech | 1 | 1 | 73 |
| ins-pop | 1 | 1 | 74 |
| ins-eco | 9 | 9 | 83 |
|  |  |  |  |
| **Three-factor causation** |  |  |  |
| pop-eco-tech | 3 | 3 | 86 |
| pop-awar-tech | 1 | 1 | 87 |
| pop-eco-awar | 1 | 1 | 88 |
| eco-awar-tour | 1 | 1 | 89 |
| ins-pop-eco | 3 | 3 | 91 |
| ins-eco-cult | 2 | 2 | 93 |
| ins-eco-tech | 2 | 2 | 95 |
|  |  |  |  |
| **Four-factor causation** |  |  |  |
| pop-eco-tech-tour | 1 | 1 | 96 |
| ins-pop-eco-tech | 1 | 1 | 97 |
| ins-pop-eco-awar | 2 | 2 | 99 |
|  |  |  |  |
| **Five-factor causation** |  |  |  |
| ins-pop-eco-awar-tech | 1 | 1 | 100 |
|  |  |  |  |
| **Total** | 105 | 100 | - |
