# Supplementary material for: Drivers of Wetland Conversion: a Global Meta-Analysis
Source: PLoS One. 2013 Nov 25;8(11):e81292. doi: 10.1371/journal.pone.0081292 (PMC3840019; doi:10.1371/journal.pone.0081292)
Supplement: Information S7 — Results of a frequency analysis including only the cases since 1980. (DOCX) [file pone.0081292.s008.docx]

**Supporting Information S8**

Results of a frequency analysis including only the cases since 1980.

| **Proximate causes** | | | |
| --- | --- | --- | --- |
| Agricultural development | 19 | Arable land | 12 |
|  |  | Aquaculture | 5 |
|  |  | Harvesting for bioresources (cranberries, reed cutting, medicinal plants) | 0 |
|  |  | Plantations | 2 |
|  |  | Drainage to combat malaria, dysentery, cholera | 0 |
| Wood extraction | 3 | Large-scale logging | 3 |
|  |  | Local use of wood | 0 |
| Pasture expansion | 2 | - | 2 |
| Settlement development | 10 | Urban | 9 |
|  |  | Rural | 1 |
| Industrial/commercial development | 8 | Oil and gas extraction | 0 |
|  |  | Water extraction (for e.g. irrigation, hydroelectical power) | 2 |
|  |  | Industrial/commercial activities/extraction: other | 6 |
| Peat extraction | 2 | For fuel (local use versus large-scale peat extraction) | 2 |
|  |  | For horticulture/litter/fertilizer | 0 |
| Infrastructure construction | 8 | Roads, airport, recreational functions, etc. | 4 |
|  |  | Dam construction / Reservoirs (sediment deficit) | 2 |
|  |  | Canal dredging/filling/dykes (for land drainage) / boat traffic | 2 |
| Natural causes | 7 | Climatic events: droughts, fires, storms, cyclones | 1 |
|  |  | Increased temperature | 3 |
|  |  | Decreased precipitation | 2 |
|  |  | Sea-level rise | 1 |
| **Underlying factors** | | | |
| Institutional factors (mainly government regulations and subsidies) | | | 6 |
| Population growth | | | 13 |
| Economic growth / benefits | | | 16 |
| Little environmental awareness | | | 0 |
| Technological innovations | | | 5 |
| Cultural reasons (traditional use of the land by local people) | | | 0 |
| Tourism/recreational developments | | | 0 |
