# Supplementary material for: Drivers of Wetland Conversion: a Global Meta-Analysis
Source: PLoS One. 2013 Nov 25;8(11):e81292. doi: 10.1371/journal.pone.0081292 (PMC3840019; doi:10.1371/journal.pone.0081292)
Supplement: Information S8 — Logistic regression using counterfactual locations based on the Protected Planet database. (DOCX) [file pone.0081292.s009.docx]

**Supporting Information S9**

The logistic regression was repeated (same method as described in the paper) using four data sets of non-converted wetlands that were generated randomly based on the Protected Planet database (protectedplanet.net). The principal component analysis resulted in the same patterns (Table S9.1). Therefore, the same set of parameters was selected for the (stepwise) regression analyses. Regression results show that market influence is the most important and statistically significant in all cases except for one (Table S9.2 and S9.3). This indicates the overriding influence of this factor. Built-up area, cropland area, population density and temperature were also significant in several cases, corresponding to the analyses using non-converted data sets generated based on the Global Lakes and Wetland Database. The main difference between the two analyses is that in the ‘Protected Planet analysis’ precipitation is statistically significant in most cases (6 out of 8) and wetland area is never significant, while in the ‘GLWD analysis’ precipitation is never significant and wetland area is significant in all cases. This may be explained by the different locations of the non-converted wetland sites (protected wetlands may be found at specific locations with specific conditions) and/or by determinants not included in the analyses (related to these specific locations). Also, the average and median wetland area for all non-converted wetland site in the ‘GLWD analysis’ (0.80 and 0.89 respectively) is higher compared to the average and median wetland area for all non-converted wetland site in the ‘Protected Planet analysis’ (0.67 and 0.78 respectively). This might be one of the explanations of the greater importance of wetland area in the ‘GLWD analysis’. Overall, the replacement of precipitation by annual temperature as explanatory variable and the inclusion of adjacent prevalence of wetlands only make the pattern more plausible.

**Table S9.1. Principal components loadings.** All wetland conversion cases and the no wetland conversion sites are included in the PCA analysis (*N*=213). The percentage of variance explained by each component is indicated between brackets.

|  | **Principal Component** | | | |
| --- | --- | --- | --- | --- |
|  | **1 (24%)** | **2 (21%)** | **3 (10%)** | **4 (10%)** |
| Distance to roads | 0.42 | -0.46 | -0.07 | -0.01 |
| Wetland area | 0.16 | -0.45 | -0.04 | 0.12 |
| Rule of law | 0.90 | 0.34 | -0.05 | -0.07 |
| Government effectiveness | 0.90 | 0.31 | -0.01 | -0.08 |
| Regulatory quality | 0.88 | 0.37 | 0.00 | -0.03 |
| Voice and accountability | 0.90 | 0.25 | 0.02 | -0.07 |
| Percentage of Histosols | 0.22 | -0.33 | 0.86 | 0.01 |
| Slope | -0.09 | 0.13 | 0.00 | 0.17 |
| Market accessibility | -0.08 | 0.83 | 0.06 | 0.23 |
| Market influence | -0.02 | 0.84 | 0.15 | -0.05 |
| Built-up area | 0.05 | 0.52 | -0.04 | 0.63 |
| Population density | -0.14 | 0.37 | 0.03 | 0.48 |
| Cropland area | -0.38 | 0.52 | 0.17 | -0.59 |
| Efficiency of agricultural production | -0.28 | 0.52 | 0.08 | -0.68 |
| Soil organic content | 0.29 | -0.30 | 0.84 | 0.00 |
| Precipitation | -0.29 | 0.26 | 0.41 | 0.23 |
| Temperature | -0.50 | 0.33 | 0.28 | 0.17 |

**Table S9.2. Independent variables explaining the occurrence of wetland conversion for four different datasets.** The sign of each significant variable is indicated with ‘+’ and ‘-‘. Back = backward selection method and For = forward selection method (Probability for stepwise selection: P_in_=0.01, P_out_=0.02).

|  | **Dataset 1** | | **Dataset 2** | | **Dataset 3** | | **Dataset 4** | |
| --- | --- | --- | --- | --- | --- | --- | --- | --- |
| Selection method | Back | For | Back | For | Back | For | Back | For |
| Slope |  |  |  |  |  |  |  |  |
| Temperature |  | + |  |  |  |  | + | + |
| Precipitation |  |  | + | + | + | + | + | + |
| Market influence |  | + | + | + | + | + | + | + |
| Population density |  |  | + |  | + |  |  |  |
| Built-up area | + |  |  |  |  |  | + |  |
| Distance to roads | - |  | - |  |  |  |  |  |
| Wetland area |  |  |  |  |  |  |  |  |
| Cropland area | + |  |  |  | + | + | + | + |
| Organic content |  |  |  |  |  |  |  |  |
| Regulatory Quality |  |  |  |  |  |  |  |  |
| ROC | 0.821 | 0.804 | 0.877 | 0.842 | 0.904 | 0.886 | 0.892 | 0.884 |

**Table S9.3.** **Regression coefficients (and Standard Error: ±) and ROC values for the 4 datasets**, using precipitation, market access, population density and cropland in the neighbourhood as independent variables to explain the occurrence of wetland conversion.

|  | **Constant** | **Precipitation** | **Market influence** | **Population density** | **Cropland area** | **ROC** |
| --- | --- | --- | --- | --- | --- | --- |
| **Dataset 1** | -2.273 ± 0.394 | 0.007 ± 0.003 | 0.403 ± 0.115 | 0.003 ± 0.011 | 1.769 ± 0.815 | 0.827 |
| **Dataset 2** | -2.627 ± 0.399 | 0.012 ± 0.003 | 0.493 ± 0.118 | 0.003 ± 0.012 | 1.695 ± 0.893 | 0.870 |
| **Dataset 3** | -3.049 ± 0.452 | 0.012 ± 0.003 | 0.545 ± 0.126 | 0.005 ± 0.002 | 2.955 ± 1.082 | 0.904 |
| **Dataset 4** | -2.652 ± 0.398 | 0.010 ± 0.003 | 0.595 ± 0.117 | 0.001 ± 0.001 | 2.635 ± 0.930 | 0.864 |
